# Exploring the Indications, Levels, and Outcomes of Lower Extremity Amputation at a Tertiary Care Hospital in Pakistan

**DOI:** 10.7759/cureus.48856

**Published:** 2023-11-15

**Authors:** Malik Amna Khatoon, Syed Muhammad Khalid Karim, Laraib Khan, Sundas Karimi, Umar Farooq Abro

**Affiliations:** 1 Orthopedic Surgery, Dow University of Health Sciences, Dow International Medical College, Karachi, PAK; 2 Orthopedics and Trauma, Dow University of Health Sciences, Dow International Medical College, Karachi, PAK; 3 Medical Education, Dow University of Health Sciences, Dow International Medical College, Karachi, PAK; 4 Orthopedics, Liaquat National Hospital and Medical College, Karachi, PAK

**Keywords:** diabetes mellitis, major limb amputation, endocrinology and diabetes, pakistan, diabetic foot ulcers, lower extremity amputation

## Abstract

Background: Lower extremity amputation (LEA) is a surgical procedure performed to remove either a part or the entire lower limb due to medical conditions such as trauma, infection, peripheral vascular disease, or malignancy. The procedure is becoming increasingly common in Pakistan, with a bulk of patients presenting from rural areas in tertiary care centers. Understanding the indications, levels, and outcomes of LEA is essential for improving patient care and adopting preventive strategies, especially in developing countries.

Methodology: This study was conducted at Dow University Hospital in Karachi, Pakistan. Retrospective data of 384 patients who underwent non-traumatic lower extremity amputations between January 2016 and December 2020 was collected to include relevant history and characteristics, amputation indication and level, type of anesthesia used, and outcome within hospital stay. The data was analyzed using descriptive statistics.

Results: The data is composed of a wide age range (18 to 91 years) of patients, including a male majority (76.3%, n = 293). The employment status of the patients was taken into consideration, with a reported high number of unemployed individuals (60.4%, n = 232). Diabetes mellitus (84.4%, n = 324) was a commonly reported past medical condition, followed by hypertension (4.4%, n = 17). Indications for amputation exceedingly recorded were diabetic foot ulcers (84.4%, n = 324), followed by infections (9.4%, n = 36) and peripheral arterial disease (3.6%, n = 14). The anesthetic approach that was observed most in these patients was regional anesthesia (74.7%, n = 287). Right-sided amputations (52.9%, n = 203) were dominant, with below-knee amputations leading by the level of amputation performed (42.5%, n = 163). Many patients delayed seeking treatment (71.6%, n = 275) and indicated denial of severity (18%, n = 69) as a reason for the delay. Regarding outcome, many patients were successfully discharged following treatment (85.9%, n = 330).

Conclusion: Overall, LEAs are being frequently performed in developing countries, such as Pakistan, especially with a large population living with diabetes mellitus. The implications of this disease are reflected in this study population, with the majority of patients reporting delays in treatment due to reasons such as the unknown severity of the disease or financial burdens. The challenges faced by these individuals, especially in this country, can be tackled with widespread affordability and availability of care and education on early management.

## Introduction

Amputation involves removing a body part, and LEA procedures typically involve the removal of a part or the whole limb following trauma or damage due to underlying medical causes. Non-traumatic causes of amputations are rising with diabetes mellitus at the backdrop of these procedures. Even in regions with structured healthcare and standardized guided treatment for foot disease, amputation is inevitable in patients with worsening diabetes, especially older males [1]. Data from the last 60 years of foot diseases in African countries has shown diabetic foot lesions have increased, contributing to high mortality around limited health services [2].

The overall burden of LEA is global, with most populous countries reporting a rise in otherwise preventable diseases leading to these procedures. For instance, in India, a 54.1% increase was noted in major lower limb amputations, largely due to uncontrolled diabetes or peripheral artery disease, leading to a severe impact on the quality of life of patients [3].

A stigma is also associated with such procedures, and so patients refrain from getting timely care to prevent amputation. This leads to performing a major procedure for what would have been a minor problem. In Hong Kong, a study reported patient discrimination against LEA, as it is considered a taboo topic, so much so that patients needing lifesaving amputations avoid intervention entirely [4].

Pakistan, being a developing country, is faced with continuous setbacks in disease prevalence due to the majority of the population living in rural areas with little to no access to healthcare. The country is also facing substantial LEA rates with an increase in the population of diabetic patients. According to the International Diabetes Federation, Pakistan in 2021 had an estimated 30.8% of the population with diagnosed diabetes and 26.9% of the population with undiagnosed diabetes, compared to the United States at 10.7% diagnosed and 12.5% undiagnosed diabetes [05]. In addition to limited healthcare services, the majority of the Pakistani population lives well below their means and ignores healthcare intervention until it is necessary. There is limited data available on rates of LEA in Pakistan, but given the percentage of increasing diabetes, amputation rates would be notable.

Infrastructure plays a big role in shaping the healthcare foundation of a country. Accessible care is needed in Pakistan as it is encountering excessive LEA rates with what could be preventable or treatable conditions. The present study aims to investigate causes of LEA at a tertiary care hospital in Pakistan and contribute to understanding aspects associated with demographics, levels, and delays of these procedures.

## Materials and methods

Data for this retrospective cohort study was collected at the Dow University of Health Sciences in Karachi, Pakistan. The study included all patients who had undergone non-traumatic LEA between January 2016 and December 2020, with a sample size consisting of 384 patients. Patients who had a traumatic lower limb amputation were excluded from the study. Patients with incomplete medical records were also excluded from the study.

Data from medical records was utilized to gather information on patient demographics, including age, gender, and occupation. The comorbidities observed were indicated either individually or grouped if the patient had multiple ailments. Those with no comorbidities were also grouped separately. Indications for amputation were categorized as diabetic foot ulcer, infection, lower limb deformity, malignancy, or peripheral arterial disease. Surgical notes on the side, the level of amputation performed, and the type of anesthesia given were requested.

A comprehensive review of the patient's history was carried out to identify the reason for the delay in treatment, if any. A delay in treatment was indicated as “yes” if the patient was either unable to seek appropriate treatment or refused earlier treatment on personal grounds. This delay could be anytime, from the onset of symptoms to the presentation at the hospital. Reasons for delay given were denial of severity, fear of amputation, financial issues, herbal treatment, a lack of facilities nearby, or self-treatment. A “no” in surgical delay meant the patient had amputation performed after failed preventive measures. The average length of hospital stay was determined, and only patient outcomes during the duration of the stay were noted. Outcomes were recorded as discharged, expired, leave against medical advice, or referred to another facility.

Strict measures were implemented to ensure the privacy and confidentiality of patient information throughout the study. Ethical clearance for the study was obtained from the Institutional Review Board of Dow University of Health Sciences (approval no. IRB-3110/DUHS/EXEMPTION/2023/285). Considering the large sample size of patients involved, this was a retrospective study utilizing existing data with no active involvement of patients; therefore, informed consent was not required.

The collected data was entered into Microsoft Excel (Microsoft Corp., Redmond, WA, USA). All statistical analyses were conducted using SPSS Statistics version 20 (IBM Corp., Armonk, NY, USA). Descriptive statistics were utilized to present the fundamental demographic characteristics, including the mean, standard deviation, and range for continuous variables, while frequencies and proportions were used for categorical variables. A significance level of p < 0.05 was considered statistically significant. The obtained results are appropriately grouped in tables.

## Results

A total of 384 individuals were operated on for lower limb amputation, covering a diverse age spectrum from 18 to 91 years, with an average age of 59.70 years (standard deviation = 12.641). Among the individuals included in the study, a majority of 76.3% (n = 293) were male, whereas 23.7% (n = 91) were female. The occupational profiles were classified into five distinct groups. Out of the total sample size, it was found that 60.4% (n = 232) were unemployed, 19.8% (n = 76) were self-employed, 9.9% (n = 38) were government employees, 8.9% (n = 34) were employed in the private sector, and 1.0% (n = 4) were enrolled as students (Table 1).

**Table 1:** Demographic characteristics

Characteristics		Frequency (n)	Percentage
Gender	Female	91	23.7
	Male	293	76.3
	Total	384	100.0
		Frequency (n)	Percentage
Occupation	Government	38	9.9
	Private	34	8.9
	Self-employed	76	19.8
	Student	4	1.0
	Unemployed	232	60.4
	Total	384	100.0

The most prevalent historical medical ailment reported was diabetes mellitus, as indicated by 84.4% (n = 324) of the subjects (Table 2). The prevalence of hypertension was seen in 4.4% (n = 17) of the study population. Additional comorbidities seen in the study population were chronic kidney disease in 1.3% (n = 5) of individuals, chronic obstructive pulmonary disease in 1.0% (n = 4) of individuals, and ischemic heart disease in 1.0% (n = 4) of individuals.

**Table 2:** Frequency of various comorbidities CKD: Chronic kidney disease, HTN: Hypertension, IHD: Ischemic heart disease, COPD: Chronic obstructive pulmonary disease, DM: Diabetes mellitus

Comorbidity	Frequency (n)	Percentage
Asthma	3	0.8
CKD	5	1.3
HTN	17	4.4
IHD	4	1.0
COPD	4	1.0
Dementia	2	0.5
DM	324	84.4
Individuals with no comorbidities	18	4.6
Multiple comorbidities		
CKD, HTN, IHD	1	0.3
COPD, IHD	1	0.3
DM, Dementia	1	0.3
DM, IHD	1	0.3
HTN, CKD	1	0.3
HTN, IHD	2	0.5
Total	384	100.0

The leading cause of LEAs was found to be diabetic foot ulcers, which constituted 84.4% (n = 324) of the observed cases (Table 3). Infection was identified as the second most common cause, accounting for 9.4% (n = 36) of the study participants. The prevalence of lower limb malformation in the amputations was found to be 1.8% (n = 7), while malignancy accounted for 0.8% (n = 3) of the cases. A few individuals (3.6%, n = 14) were found to have peripheral artery disease as the cause of amputation. These were patients who were diagnosed elsewhere with this condition and came to our institution with a referral for amputation.

**Table 3:** Causes of amputation

Causes	Frequency (n)	Percentage
Diabetic foot ulcer	324	84.4
Infection	36	9.4
Lower limb deformity	7	1.8
Malignancy	3	0.8
Peripheral arterial disease	14	3.6
Total	384	100.0

The predominant form of anesthesia given was regional anesthesia, accounting for 74.7% (n = 287) of the cases (Table 4). General anesthesia was the second most common type, comprising 13.3% (n = 51) of the cases, while local anesthesia was administered in 9.9% (n = 38) of the cases. The distribution of amputation sites in the study population was as follows: 52.9% (n = 203) of cases included right-side amputations, 45.8% (n = 176) involved left-side amputations, and bilateral amputations were observed in 1.3% (n = 5) of cases (Table 4). Below-knee amputation was the most frequently performed level of amputation at 42.4% (n = 163), followed by ray amputation at 18.2% (n = 70) and toe amputation at 27.3% (n = 105) (Table 4).

**Table 4:** Surgical aspects

Parameters		Frequency (n)	Percentage
Type of anesthesia	Block	4	1.0
	General	51	13.3
	Local	38	9.9
	Regional	287	74.7
	Sedation	4	1.0
	Total	384	100.0
Side of amputation	Bilateral	5	1.3
	Left	176	45.8
	Right	203	52.9
	Total	384	100.0
Level of amputation	Hip disarticulation	3	0.8
	Above knee amputation	33	8.6
	Below knee amputation	163	42.5
	Forefoot amputation	10	2.6
	Ray amputation	70	18.2
	Toe amputation	105	27.3
	Total	384	100.0

Delay in treatment was seen in many patients (71.6%, n = 275), with reasons provided such as denial of the severity of the disease (18.0%, n = 69) or financial burden (16.4%, n = 63) (Table 5). However, 28.4% (n = 109) of patients did not encounter any form of delay.

**Table 5:** Analysis of delays in amputation surgery

Variable	Response	Frequency (n)	Percentage
Delay in surgery	No	109	28.4
	Yes	275	71.6
	Total	384	100.0
Reason of delay	Denial of severity	69	18.0
	Fear of amputation	38	9.9
	Finance	63	16.4
	Herbal treatment	35	9.1
	Lack of facility nearby	29	7.5
	Self-treatment	41	10.7
	No delay in treatment	109	28.4
	Total	384	100.0

The overall outcome showed that 85.9% (n = 330) of patients were discharged, while a small percentage (5.2%, n = 20) expired during their hospital stay (Table 6). Few patients (4.2%, n = 16) abandoned care and left against medical advice. Furthermore, 4.7% (n = 18) of patients were transferred to alternative healthcare facilities for further treatment due to their additional ailments.

**Table 6:** Patient outcome after amputation LAMA: Leave against medical advice

Outcomes	Frequency (n)	Percentage
Discharged	330	85.9
Expired	20	5.2
LAMA	16	4.2
Referred	18	4.7
Total	384	100.0

The length of stay varied per patient according to the severity of co-morbidities, condition on arrival, and any postoperative complications. The patients in the study had a mean length of hospital stay of 4.97 days, with a range spanning from two to 34 days (Table 7).

**Table 7:** Length of hospital stay

	Total	Minimum (days)	Maximum (days)	Mean	Standard deviation
Length of Stay	384	2	34	4.97	3.541

A cross-tabulation was performed to analyze the association between the cause of amputation and the resulting outcome (Table 8). It presents the frequency distribution of outcome categories for each reason for amputation, along with the corresponding p-value.

**Table 8:** Association of cause and outcome of amputation LAMA: Leave against medical advice

Cause of amputation	Discharged (n)	Expired (n)	LAMA (n)	Referred (n)	p-value
Diabetic foot ulcer	296	9	9	10	0.00
Infection	18	7	7	4	
Lower limb deformity	5	0	0	2	
Malignancy	1	2	0	0	
Peripheral arterial disease	10	2	0	2	
Total	330	20	16	18	

Within the cohort of 384 individuals, the distribution of causes for amputation among different age groups was also analyzed (Table 9). A significant number of individuals, aged over 55 years, presented with diabetic foot ulcers. The p-value quantifies the level of statistical significance pertaining to the relationship between different age groups and the factors contributing to amputation.

**Table 9:** Association of age and cause of amputation

Age (in years)	Cause of amputation					
	Diabetic foot ulcer (n)	Infection (n)	Lower limb deformity (n)	Malignancy (n)	Peripheral arterial disease (n)	p-value
15-35	3	3	1	0	0	0.027
36-55	127	9	3	1	2	
56-75	160	19	3	2	10	
76-95	34	5	0	0	2	
Total	324	36	7	3	14	

## Discussion

Lower extremity amputation is becoming quite common in Pakistan, and a lack of health resources could be a crucial factor. Diabetes has triumphed as the major cause of these procedures, as indicated in the results. Diabetic foot problems, without routine management of wounds, have significantly contributed to rising amputation rates worldwide [6].

Peripheral arterial disease is caused by impaired blood flow to limbs and, if not treated promptly, is another cause of amputation, as reported in many studies. Endovascular and re-vascular surgeries have decreased the rate and level of amputations in Western countries, while infections have taken over as the cause of major amputations in countries such as Togo [7].

A few individuals presented with peripheral artery disease, but these patients were referred from elsewhere after taking a vascular surgery opinion. The limited presentation of peripheral artery disease may be due to under-reporting or under-diagnosis, particularly among rural patients who have challenges accessing diagnostic resources. The lack of a vascular surgery team at our institution prior to 2022 was also identified as a contributing factor [8].

The socio-demographic analysis revealed a diverse age range among the study participants, with an average age of 59.70 years. Lower extremity amputations are more common in older individuals, as noted in previous studies, with an age range of 65 to 74 years [09]. Looking at our analysis of age with cause of amputation, individuals between 56 and 75 years of age had the greatest number of amputations performed, with diabetes as the main cause. Additionally, many of the patients were male, which is consistent with the higher prevalence of amputations in males reported in previous studies [10]. Recently, there has been an elevated incidence of diabetes mellitus among the male population in Pakistan, considering many factors like lifestyle choices, family history, and healthcare-seeking patterns [11]. Regardless of the reason, preventable causes have taken over amputation rates in low or middle-income countries, with most patients aged under 40 years, while patients in high-income countries suffer limb loss mainly after the sixth decade of life [12].

As observed in this study, diabetes mellitus was the most prevalent medical condition, affecting 84.4% of the participants. This establishes the association between diabetes and lower extremity amputations [13]. Hypertension, chronic kidney disease, chronic obstructive pulmonary disease, and ischemic heart disease were also identified as co-existing conditions in a small proportion of participants. The presence of multiple comorbidities and complex medical histories can impact the outcomes and management of individuals undergoing LEA [14]. The primary cause of LEA was a diabetic foot ulcer, which is consistent with the number of individuals presenting with diabetes mellitus. Previous studies have emphasized the critical role of foot ulcers in diabetic patients, leading to amputations [15-17]. For this reason, early detection and appropriate management of diabetic foot ulcers are needed to prevent amputations [18,19]. An analysis of patients presenting with foot ulcers can be advantageous in such studies by identifying these ulcers according to classification and determining their etiology to detect the proper frequency of the types of ulcers presenting in the above demographic. Infections and lower limb deformities were also noted to contribute to the amputation cases, indicating a potential disability burden in the country. A more in-depth analysis could be beneficial for these individuals to identify the type of infection and background of the lower limb deformity.

Regarding surgical aspects, regional anesthesia was the most common type of anesthesia used during the amputation procedures. No relation was observed between the type of anesthesia and outcome, but regional anesthesia has been shown to provide effective pain control and improved patient satisfaction in LEAs [20]. Below-knee amputation was the most frequently performed level of amputation, followed by toe and ray amputations. A valuable idea would have been to classify wound grade and its association with the level of amputation. Further wound grade analysis is beneficial to identify patient outcomes, as the classification of wound and amputation risk has been mainly disregarded in many studies [21].

It is concerning that a significant proportion of participants experienced a delay in undergoing amputation surgery, with reasons ranging from denial of severity to financial constraints. This is also in relation to the vast number of patients who reported being unemployed. The variables contributing to treatment delays in Pakistan, with a particular focus on global concerns, are associated with infrastructure, economics, and a healthcare system that restricts patients' ability to receive timely care [22]. Delays in amputation surgery can lead to complications, prolonged hospital stays, and increased morbidity and mortality. Efforts should be made to address these barriers and ensure timely access to surgical interventions for individuals requiring LEA, especially where healthcare access is limited [23-25].

In terms of patient outcomes, the majority of participants were discharged after the amputation procedure, while a small percentage expired or left against medical advice. A follow-up for these individuals in terms of rehabilitation could help in optimizing patient outcomes and minimizing postoperative complications [26]. The mean length of hospital stay was 4.97 days, and most patients who were discharged were deemed fit with no wounds or health concerns. This average length of stay is consistent with previous studies reporting varying lengths of hospitalization following a timely discharge for LEA patients [27].

Screening and intervention of at-risk patients would largely eliminate the problems associated with foot ulceration caused by diabetes or triggered by peripheral neuropathy, peripheral artery disease, or foot deformities [28]. All these factors ultimately require amputation. If treated in time, as observed in the study from Finland, the level of amputation decreased from proximal levels to toes, and immediate limb revascularizations avoided amputations entirely [29].

Multidisciplinary diabetic foot and wound care teams, patient counseling, and community awareness programs have led to reduced amputation rates, according to multi-center evaluations conducted in Nigeria [30]. Therefore, it is all dependent upon access to healthcare facilities, timely intervention, and follow-up care that can prevent the need for LEA.

A few limitations can be noted in this research. First, the data were collected from a single center, which may limit the generalizability of the findings. Second, the sample size was relatively small, which could impact the statistical power and precision of the results. Future studies with larger sample sizes and multi-center designs are warranted to validate and expand on these findings.

## Conclusions

Lower extremity amputations are becoming common in developing countries such as Pakistan, and more studies are needed to establish well-defined actions for improvement. Patients with limited access to care and knowledge of their condition are a vital factor in the continued increase in amputations. The findings of this study provide valuable insights into the socio-demographic characteristics, past medical conditions, clinical characteristics, surgical aspects, causes for delays, and outcomes among individuals undergoing LEA. Understanding these factors is crucial for optimizing treatment approaches and improving patient care in this population.

## References

[1] Rosien L, van Dijk PR, Oskam J, Pierie ME, Groenier KH, Gans RO, Bilo HJ (2023). Lower extremity amputation rates in people with diabetes mellitus: a retrospective population-based cohort study in Zwolle Region, The Netherlands. Eur J Vasc Endovasc Surg.

[2] Abbas ZG, Boulton AJ (2022). Diabetic foot ulcer disease in African continent: 'from clinical care to implementation' - Review of diabetic foot in last 60 years - 1960 to 2020. Diabetes Res Clin Pract.

[3] Viswanathan V, Nachimuthu S (2023). Major lower-limb amputation during the COVID pandemic in South India. Int J Low Extrem Wounds.

[4] Yip K, Yip Y, Tsui W (2023). Thoughts and experiences regarding leg amputation among patients with diabetic foot ulcers: a phenomenological study. Int Wound J.

[5] IDF (International Diabetes Federation) Diabetes Atlas. . https://diabetesatlas.org/data/en/compare/150-211/idf-country-data-comparision.html (2023). Pakistan compare with United States of America. https://diabetesatlas.org/data/en/compare/150-211/idf-country-data-comparision.html.

[6] Nouira S, Ach T, Bellazreg F, Ben Abdelkrim A (2023). Predictive factors for lower limb amputation in type 2 diabetics. Cureus.

[7] Kouevi-Koko TE, Amouzou KS, Sogan A, Apeti S, Dakey YE, Abalo A (2023). Lower extremity amputations (LEAs) in a tertiary hospital in Togo: a retrospective analysis of clinical, biological, radiological, and therapeutic aspects. J Orthop Surg Res.

[8] Firnhaber JM, Powell CS (2019). Lower extremity peripheral artery disease: diagnosis and treatment. Am Fam Physician.

[9] Mayfield JA, Reiber GE, Maynard C, Czerniecki JM, Caps MT, Sangeorzan BJ (2000). Trends in lower limb amputation in the Veterans Health Administration, 1989-1998. J Rehabil Res Dev.

[10] Hicks CW, Selvin E (2019). Epidemiology of peripheral neuropathy and lower extremity disease in diabetes. Curr Diab Rep.

[11] Adnan M, Aasim M (2020). Prevalence of type 2 diabetes mellitus in adult population of Pakistan: a meta-analysis of prospective cross-sectional surveys. Ann Glob Health.

[12] Grudziak J, Mukuzunga C, Melhado C, Young S, Banza L, Cairns B, Charles A (2019). Etiology of major limb amputations at a tertiary care centre in Malawi. Malawi Med J.

[13] Lavery LA, Armstrong DG, Wunderlich RP, Mohler MJ, Wendel CS, Lipsky BA (2006). Risk factors for foot infections in individuals with diabetes. Diabetes Care.

[14] Fortington LV, Rommers GM, Postema K, van Netten JJ, Geertzen JH, Dijkstra PU (2013). Lower limb amputation in Northern Netherlands: unchanged incidence from 1991-1992 to 2003-2004. Prosthet Orthot Int.

[15] Hingorani A, LaMuraglia GM, Henke P (2016). The management of diabetic foot: a clinical practice guideline by the Society for Vascular Surgery in collaboration with the American Podiatric Medical Association and the Society for Vascular Medicine. J Vasc Surg.

[16] Walicka M, Raczyńska M, Marcinkowska K, Lisicka I, Czaicki A, Wierzba W, Franek E (2021). Amputations of lower limb in subjects with diabetes mellitus: reasons and 30-day mortality. J Diabetes Res.

[17] Hardy DM, Lyden SP (2019). The majority of patients have diagnostic evaluation prior to major lower extremity amputation. Ann Vasc Surg.

[18] Noor S, Khan RU, Ahmad J (2017). Understanding diabetic foot infection and its management. Diabetes Metab Syndr.

[19] Matijević T, Talapko J, Meštrović T, Matijević M, Erić S, Erić I, Škrlec I (2023). Understanding the multifaceted etiopathogenesis of foot complications in individuals with diabetes. World J Clin Cases.

[20] Mufarrih SH, Qureshi NQ, Schaefer MS (2021). Regional anaesthesia for lower extremity amputation is associated with reduced post-operative complications compared with general anaesthesia. Eur J Vasc Endovasc Surg.

[21] Mills JL Sr, Conte MS, Armstrong DG, Pomposelli FB, Schanzer A, Sidawy AN, Andros G (2014). The Society for Vascular Surgery Lower Extremity Threatened Limb Classification System: risk stratification based on wound, ischemia, and foot infection (WIfI). J Vasc Surg.

[22] Basharat S, Shaikh BT, Rashid HU, Rashid M (2019). Health seeking behaviour, delayed presentation and its impact among oral cancer patients in Pakistan: a retrospective qualitative study. BMC Health Serv Res.

[23] Garg A, Lavian J, Lin G, Sison C, Oppenheim M, Koo B (2017). Clinical characteristics associated with days to discharge among patients admitted with a primary diagnosis of lower limb cellulitis. J Am Acad Dermatol.

[24] Shamir S, Schwartz Y, Cohen D, Bdolah-Abram T, Yinnon AM, Wiener-Well Y (2022). The timing of limb amputation in nontraumatic patients: impact on mortality and postoperative complication rates. J Foot Ankle Surg.

[25] Agarwal P, Kukrele R, Sharma D (2023). Delayed revascularization of extremities following vascular injuries: challenges and outcome. J Orthop.

[26] Imam B (2017). Incidence and rehabilitation of lower limb amputation in Canada, and feasibility of a novel training program. UBC.

[27] Fashandi A, Johnston LE, Upchurch GU (2016). Factors affecting length of stay and discharge needs after lower extremity amputation. Vasc Endovascular Surgery.

[28] Bus SA, Lavery LA, Monteiro-Soares M, Rasmussen A, Raspovic A, Sacco IC, van Netten JJ (2020). Guidelines on the prevention of foot ulcers in persons with diabetes (IWGDF 2019 update). Diabetes Metab Res Rev.

[29] Ponkilainen VT, Vuorlaakso M, Kaartinen I (2022). The development of lower limb amputations in Finland from 1997 to 2018: a nationwide retrospective registry study. Eur J Vasc Endovasc Surg.

[30] Ugwu E, Adeleye O, Gezawa I, Okpe I, Enamino M, Ezeani I (2019). Predictors of lower extremity amputation in patients with diabetic foot ulcer: findings from MEDFUN, a multi-center observational study. J Foot Ankle Res.

